# Draft Genome Sequences from Two Gram-Negative Bacteria, *Serratia* sp. Strain EWG9 and *Leclercia* sp. Strain EMC7, Isolated from the Earthworm Eisenia fetida

**DOI:** 10.1128/mra.00939-21

**Published:** 2022-02-17

**Authors:** Partha Barman, Nibendu Mondal, Subhajit Sen, Sumit Chatterjee, Shilpa Sinha, Wriddhiman Ghosh, Ranadhir Chakraborty

**Affiliations:** a OMICS Laboratory, Department of Biotechnology, University of North Bengal, Siliguri, West Bengal, India; b Department of Microbiology, Bose Institute, Kolkata, West Bengal, India; Indiana University, Bloomington

## Abstract

We present the draft genome sequences of two bacterial strains that are putatively unique species and belong to two different Gram-negative genera: *Serratia* sp. EWG9 and *Leclercia* sp. EMC7, recovered from the gut and cast, respectively, of the compost worm Eisenia fetida.

## ANNOUNCEMENT

Eisenia fetida is an epigeic earthworm that lives on the soil surface and feeds on the litter (1). The bacterial species richness in the gut declines as food passes through it, leading to enrichment in bacteria that can digest benzoic acid and aromatic compounds (2, 3). Bacteria that can metabolize 3-nitropropionic acid (3-NPA) were isolated from the intestines and cast of *E. fetida* in this investigation. Adult earthworms were collected from the Centre for Floriculture and Agro-Business Management, University of North Bengal (26.7072° N, 88.3554° E). They were taken to the lab and raised on a cow dung bed supplemented with plant parts of Quisqualis indica L. [which contains the anthelmintic substance (2*S*)-2-amino-3-(3,5-dioxo-1,2,4-oxadiazolidin-2-yl) propanoic acid, also known as quisqualic acid]. Worms were anesthetized with 10% ethanol, dissected to remove the whole gut, or transferred to sterile tissue paper for fresh cast collection under aseptic conditions. The gut and its contents, as well as the cast samples, were then homogenized in phosphate-buffered saline (PBS; pH 7.2). The homogenized samples were serially diluted (1:10) in PBS, pour plated in minimal agar plates (supplemented with 3-nitropropionic acid), and incubated for 72 h at 30°C. Two clonally pure bacterial cultures were isolated using this method, and they were identified as *Serratia* and *Leclercia* strains based on 16S rRNA gene sequence similarities with legitimately described *Serratia marcescens* subsp. marcescens Db11 (GenBank assembly accession: GCA_000513215.1) and *Leclercia adecarboxylata* ATCC 23216 (GenBank assembly accession: GCA_000735515.1), respectively (4). Ethical permission from the Indian Committee for the Purpose of Control and Supervision of Experiments on Animals (CPCSEA) was not required for experimentation on earthworms.

The PureLink genomic DNA isolation kit (Thermo Fisher Scientific, USA) was used to extract genomic DNA from the isolates, grown overnight in Luria broth (Himedia) at 30°C and 100 rpm. DNA was then quantified using a SPECTROstar Nano microplate reader (BMG Labtech, Germany) (5). An Ion Xpress Plus fragment library kit was used to create a library from genomic DNA that had been enzymatically sheared (Thermo Fisher Scientific). SizeSelect 2% agarose E-Gel (Thermo Fisher Scientific) was used to select around 330-bp DNA sequences, which were then sequenced on the Ion S5 system’s 540 chip (Thermo Fisher Scientific). Low-quality (>Q20) and polyclonal readings were filtered out before sequence retrieval from the Ion S5, and the adapter sequences were edited using the inbuilt PGM software (Torrent Suite 5.8.0) using default parameters: settings to filter out reads less than a minimum read length (--min-read-length of 25), adapter trimming settings (trim-adapter-cutoff of 16.0; trim-adapter-min-match of 6), and quality trimming settings (--trim-qual-window-size of 30; --trim-qual-cutoff of 16), and to filter out too-short reads (--trim-min-read-length of 25). SPAdes v3.13.0 with default parameters was used to assemble the high-quality reads into contigs (6). The National Center for Biotechnology Information (NCBI; USA) Prokaryotic Genome Annotation Pipeline (PGAP) version 4.13 (7) was used to annotate the draft genome sequences. Table 1 summarizes the results of the genomic analyses.

**TABLE 1:** Summary information on the genomes of the two new bacterial strains isolated from the gut and cast of earthworm *Eisenia fetida*

Characteristic	Data for:	
	*Serratia* sp. strain EWG9	*Leclercia* sp. strain EMC7
BioProject accession no.	PRJNA675188	PRJNA675190
SRA accession no.	SRR15400575	SRR15400871
BioSample accession no.	SAMN16691860	SAMN16691921
GenBank accession no.	JADNRO000000000	JADMNK000000000
No. of high-quality reads	7,708,601	11,512,091
Mean read length (bp)	203	202
No. of contigs	35	45
Genome size (bp)	5,040,598	5,030,213
Genome coverage (×)	274.0	593.0
GC content (%)	59.7	56.3
*N*_50_ (bp)	442,416	258,116
*L*_50_	4	5
Total no. of genes	4,787	4,747
No. of coding genes	4,544	4,507
No. of rRNAs (5S, 16S, 23S)	7, 4, 1	7, 4, 11
No. of tRNAs	79	77
No. of noncoding RNAs	15	8
No. of pseudogenes	137	133

Genome-wide analyses employing NCBI PGAP and RAST server (8) have revealed genes for benzoate degradation, putrescine usage pathways, polyamine metabolism, and aromatic amine catabolism in both genomes. The analysis revealed a gene coding for nitronate monooxygenase, a key enzyme in detoxifying 3-NPA, in the genome of *Serratia* sp. strain EWG9.

### Data availability.

The raw sequence reads were deposited to NCBI Sequence Read Archive, whereas the entire genome shotgun projects were deposited to DDBJ/ENA/GenBank (SRA). Table 1 lists all of the pertinent accession numbers.
